# Colistin Effects on Emphysematous Lung in an LPS-Sepsis Model

**DOI:** 10.3390/antibiotics12121731

**Published:** 2023-12-14

**Authors:** Rodopi Stamatiou, Anna Vasilaki, Dimitra Tzini, Konstantina Deskata, Konstantina Zacharouli, Maria Ioannou, Markos Sgantzos, Epaminondas Zakynthinos, Demosthenes Makris

**Affiliations:** 1 Physiology Laboratory, Faculty of Medicine, University of Thessaly, BIOPOLIS, 41500 Larissa, Greece; 2 Pharmacology Laboratory, Faculty of Medicine, University of Thessaly, BIOPOLIS, 41500 Larissa, Greece; a.vasilaki@uth.gr (A.V.);; 3 Intensive Care Unit, Faculty of Medicine, University of Thessaly, BIOPOLIS, 41500 Larissa, Greeceezakynth@uth.gr (E.Z.); dimomakris@uth.gr (D.M.); 4 Pathology Department, Faculty of Medicine, University of Thessaly, BIOPOLIS, 41500 Larissa, Greecemioan@uth.gr (M.I.); 5 Anatomy Department, Faculty of Medicine, University of Thessaly, BIOPOLIS, 41500 Larissa, Greece; sgantzos@uth.gr

**Keywords:** colistin, sepsis, emphysema, lung

## Abstract

Emphysema is prevalent in various respiratory diseases like Chronic Obstructive Pulmonary Disease (COPD) and cystic fibrosis. Colistin and vasoconstrictive drugs are crucial for treating these patients when diagnosed with sepsis in the ICU. This study examines colistin impact in ether-induced emphysematous septic and non-septic animals, focusing on lung pathophysiology and inflammatory responses, including IL-1β, TNF-α, AMPK, caspase-3, cyclin-D1, and colistin levels in lung tissue. All animals exhibited significant emphysematous changes, accentuated by LPS-induced septic conditions, validating the emphysema model and highlighting the exacerbating effect of sepsis on lung pathology. Colistin, alone or with vasoconstrictive drugs, stimulated immune responses through increased inflammatory cell infiltration and the presence of lymphocytes, indicating potential immunomodulatory effects. Vasoconstriction did not alter the effects of colistin or sepsis but correlated with increased colistin levels in the lungs of septic animals. These observations suggest a potential interplay between vasoconstrictive drugs and colistin distribution/metabolism, leading to enhanced local concentrations of colistin in the lung microenvironment. The findings suggest the need for further investigations to optimize colistin and vasoconstrictive drug delivery in critically ill patients with lung pathologies. Understanding these complexities may guide more effective management of inflammatory responses and lung pathologies in these critical conditions.

## 1. Introduction

Emphysema is a common pathological manifestation in various respiratory diseases, mainly Chronic Obstructive Pulmonary Disease (COPD) and cystic fibrosis [1]. One of the main characteristics of COPD is air flow limitation partly due to emphysematous alterations and loss of functional lung tissue, leading to long-term respiratory failure [2]. The main cause of COPD has been identified to be the modern way of life, with smoking being the number one factor that could lead to this chronic disease [3]. On the other hand, cystic fibrosis is a congenital disease that affects mainly the respiratory system, with emphysematous damage being evident in the lungs of patients who suffer from this disease [4]. Alveolar walls are damaged in emphysema, regardless of the cause of its manifestation. Apart from emphysema, patients suffering from either COPD or cystic fibrosis often face infections that will further worsen their respiratory condition. These infections are mainly attributed to bacteria, such as *Pseudomonas* spp. and *Stenotrophomonas maltophilia* [5]. 

Colistin belongs to the polymyxin family of antibiotics, which is renowned for its activity against Gram-negative bacteria. This antibiotic exerts its effects by binding to lipopolysaccharides (LPS) present in the outer and cytoplasmic membranes of bacterial cells. This interaction disrupts the bacterial cell membrane, resulting in a loss of structural integrity. As a consequence, the bacterium becomes more vulnerable to various forms of damage, including mechanical stress, osmotic pressure, and adverse environmental conditions [6,7]. Furthermore, colistin enables the aggregation of the circulating LPS. 

One of the primary advantages of using colistin in the treatment of bacterial infections is its efficacy against microorganisms that have developed resistance to other antibiotics [8]. As a result, colistin is often considered a last-resort option for treating infections, particularly in hospitalized patients who are at risk of multidrug-resistant infections that could progress to sepsis—a life-threatening condition associated with high mortality [9].

Colistin has been used in the treatment of infections in patients suffering from COPD and cystic fibrosis. These patients are particularly prone to infections due to compromised airway structure, impaired lung function, and altered immune profiles [10]. Respiratory infections in both COPD and cystic fibrosis patients can exacerbate the frequency and severity of disease flare-ups, further deteriorating respiratory health and hastening the progression of these conditions [10]. Additionally, the emergence of sepsis poses a significant threat to the well-being and even the survival of these patients [10]. 

Sepsis is a critical condition that is often present in patients who have been admitted to the ICUs (intensive care units) of hospitals worldwide. When managing lung infections in the ICU, especially those impacting the lower respiratory tract, it is vital to confirm that the concentration of colistin matches the severity of damage to the pulmonary tissue [11]. More specifically, the use of colistin for the treatment of ventilator-associated pneumonia (VAP) and ventilator-associated tracheobronchitis has led to the pharmacokinetic evaluation of this antibiotic in order to comprehend the variations that exist among diverse patients. These studies showed that both the concentration and the effectiveness of the drug depend on the colistin concentration in specific lung regions, notably the percentage of colistin in its active form and the presence of immune cells [11]. Colistin has also been found to interact with signaling pathways in neutrophils and alter the activity of immune cells in patients suffering from cystic fibrosis by impacting elastase activity [12,13].

Although there are studies analyzing the epidemiological data of respiratory diseases that are characterized by emphysematous alterations in the lung, as well as their clinical manifestations, the use of animal models is indispensable in clarifying the pharmacological impact of antibiotics utilized in treating these conditions. Animal models have been proven valuable for inducing emphysema and studying both its induction and potential treatments [14]. Rats are considered an excellent animal model for the study of respiratory function due to their ease of use in terms of animal breeding and drug administration. In these animals, ether inhalation is considered not only a method to induce anesthesia but also to induce emphysema, particularly when the administration is repetitive [14]. In addition, the administration of LPS intraperitoneally has been documented to be a sepsis model in adult Wistar rats [15]. One of the advantages of using an animal model is that tissue harvesting can lead to well-preserved specimens that can be assessed using different evaluation techniques for the measurement of numerous factors indicative of the activation of specific signaling pathways [16]. Hence, animal models of ether-induced emphysema and LPS-induced sepsis can be employed for the study of pharmacologic agents, like colistin, which are clinically used to treat infections in septic conditions. It is worth mentioning that sepsis is a condition known to manifest with alterations in the metabolic profile of various tissues, impacting the homeostasis of the cell cycle by activating or inhibiting the pathways involved in cell proliferation or death [17,18,19]. 

The aim of the present study was to examine the effects of colistin in the emphysematous lungs of septic and non-septic Wistar rats. The concentration of the antibiotic in the lung, the induction of inflammation and the activation of metabolic, cell death, and cell proliferation signaling pathways were also evaluated in an attempt to specify the molecular mechanisms that are responsible for the induced colistin effect. The combined use of vasoactive medications with colistin was also assessed, considering these medications are commonly utilized in clinical practice for treating hospitalized patients with sepsis. 

## 2. Results

### 2.1. Histopathological Evaluations

**Figure 1:**
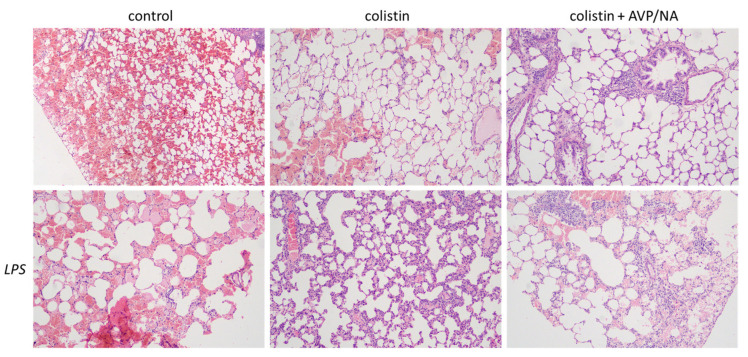
Hematoxylin-eosin staining of rat lung tissues after repetitive ether exposure. Emphysematous alterations and hemorrhage are present in all tissues studied. The presence of colistin leads to immune cell infiltration. The effect of colistin is more pronounced in the presence of LPS-induced sepsis. Vasoconstriction has no apparent effect on colistin-induced inflammatory cell lung infiltration. Abbreviations: control: ether-induced emphysematous animals; septic: LPS-induced septic animals; AVP/NA: arginine vasopressin/noradrenaline, LPS: LPS-treated septic animals. The magnification used in the microscope was 20×.

Emphysematous damage is obvious in all lung specimens. Septic animals appear to have more extensive damages than non-septic ones. Furthermore, the presence of colistin with or without vasoactive drugs induced an increase in inflammatory cell infiltration. The presence of lymphocytes is obvious in tissues from colistin-treated animals. Vasoconstriction does not seem to affect either the colistin or the sepsis effect (Figure 1). 

### 2.2. Colistin Tissue Levels

**Figure 2:**
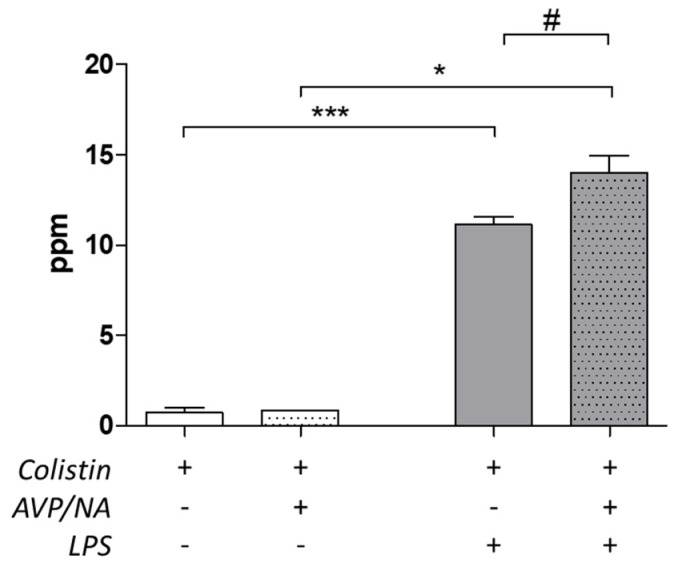
Colistin levels evaluation in emphysematous lung samples. Increased levels are observed in LPS-induced septic animals (* *p* < 0.05 and *** *p* < 0.001, Mann–Whitney test, compared to relative non-septic animals). The presence of vasoconstriction further elevates colistin levels in septic animals (# *p* < 0.05, Mann–Whitney test, LPS-induced septic colistin treated + VA-treated animals compared to LPS-induced septic colistin treated animals). Abbreviations: AVP/NA: arginine vasopressin/noradrenaline, LPS: LPS-treated septic animals.

Evaluation of colistin levels in lung tissue homogenates revealed that the levels of the antibiotic were increased in the lungs of emphysematous/septic animals. Namely, while in emphysematous/non-septic animals, the levels of the antibiotic were 0.72 ± 0.27 ppm in colistin-only and 0.85 ± 0.97 ppm colistin and vasoactive drugs treated animals, in emphysematous/septic animals colistin levels increased to 11.13 ± 0.45 ppm and 13.99 ± 0.97 ppm, respectively (* *p* < 0.05, *** *p* < 0.001, Mann–Whitney test, septic vs. non-septic animals, correspondingly treated, Figure 2). In the LPS-induced septic animals, the presence of vasoconstriction led to an increase in colistin levels in the tissue (# *p* < 0.05, Mann–Whitney test, colistin + VA vs. colistin in LPS-induced sepsis, Figure 2). 

### 2.3. Cytokine Production

**Figure 3:**
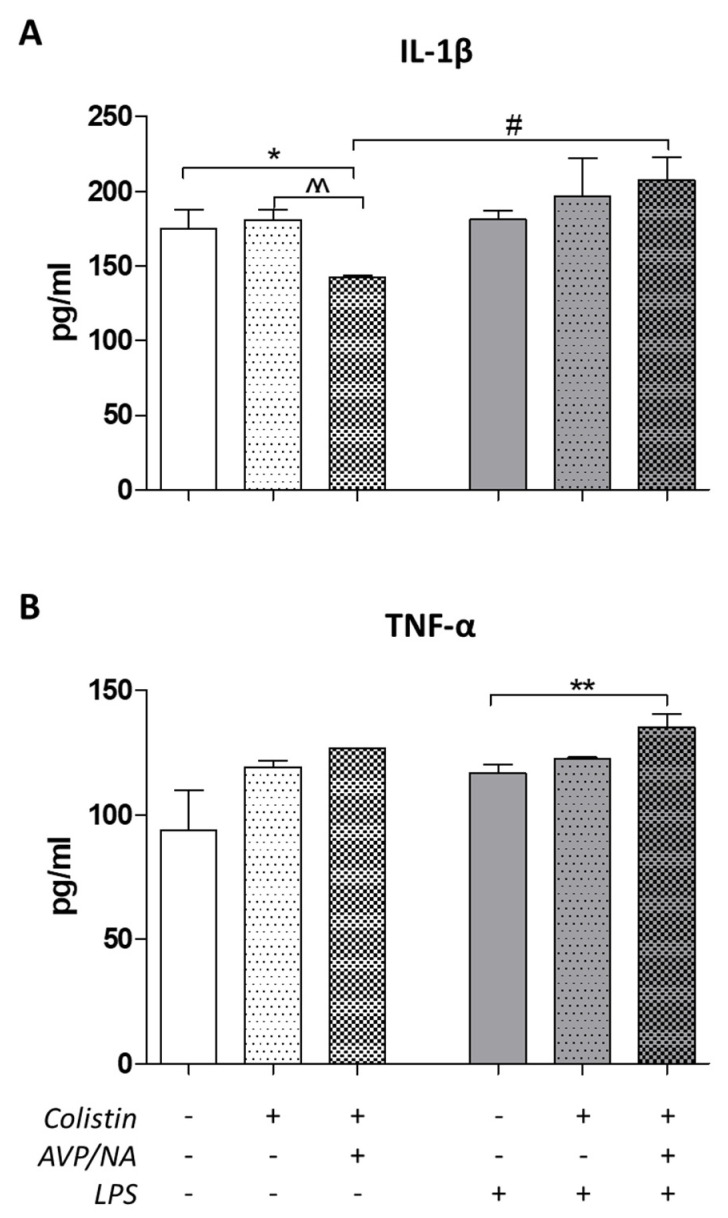
Cytokine levels in lung tissues. (**A**). IL-1β concentrations are lower in colistin + VA-treated non-septic animals compared to both control or colistin alone ones (* *p* < 0.05, Mann–Whitney test, colistin + VA compared to control, ^^ *p* < 0.01, Mann–Whitney test, colistin + VA compared to colistin). Vasoconstriction enhances IL-1β production in specimen from septic animals (# *p* < 0.05, Mann–Whitney test, colistin + VA in septic vs. non − septic animals). (**B**). The presence of vasoconstriction elevates TNF-α levels in septic animals (** *p* < 0.05, Mann–Whitney test, LPS colistin + VA compared to control).

Both IL-1β and TNF-α were detected in the lung homogenates of control/emphysematous animals, with TNF-α and IL-1β levels being 93.75 ± 16.25 pg/mL and 175 ± 12.5 pg/mL, respectively. IL-1β levels were lower in rats treated with colistin in the presence of vasoconstrictive drugs in non-septic animals (^^ *p* < 0.01, Mann–Whitney test, Figure 3A). On the other hand, in septic animals, IL-1β levels were 196.9 ± 25.16 pg/mL in the lungs of colistin-treated animals and 207.5 ± 15 pg/mL in the colistin + VA-treated ones (Figure 3A), showing that vasoactive drugs did not lower IL-1β levels like in non-septic rats. As far as TNF-α was concerned, in both non-septic and septic animals, the presence of colistin does not seem to significantly affect TNF-α levels compared to the control/emphysematous group, with the levels in non-septic control animals being 93.75 ± 16.25 pg/mL and the colistin-treated group being 119.2 ± 2.5 pg/mL, respectively. In LPS-induced septic animals, the levels were 116.9 ± 3.43 pg/mL and 122.5 ± 0.83 pg/mL in the control and colistin-treated animals, respectively (Figure 3B). As indicated in Figure 3, TNF-α levels in the lungs of colistin + VA septic animals were statistically significantly higher than in the corresponding control group (** *p* < 0.01, Mann–Whitney test, Figure 3B). 

### 2.4. Signaling Pathways Activated

**Figure 4:**
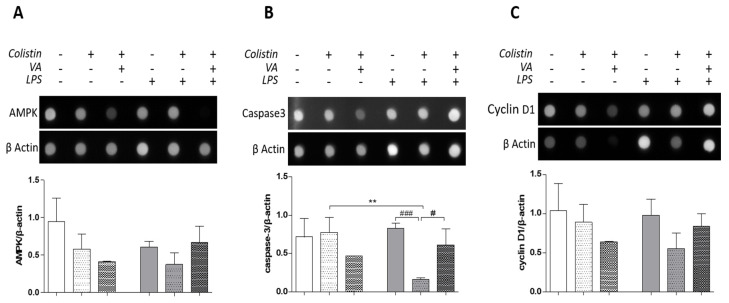
Signaling pathways activated by colistin in lung homogenates. (**A**). AMPK levels are not altered in the presence of colistin and/or sepsis in lung tissue. (**B**). Caspase-3 levels are lower in colistin-treated septic animals (** *p* < 0.01, Mann–Whitney test compared to colistin in non-septic animals, ### *p* < 0.001, Mann–Whitney test compared to control septic animals). The presence of vasoactive drugs ameliorated the colistin-induced effect on caspase-3 levels (# *p* < 0.05, Mann–Whitney test colistin + VA compared to colistin alone in septic animals). (**C**). Cyclin-D1 levels are not affected by the presence of colistin and/or sepsis.

Dot blot analysis of lung homogenates was used to evaluate changes in the metabolism, cell death and cell proliferation due to colistin treatment. Colistin alone or combined with vasoconstrictive drugs did not have any statistically significant effect on AMPK and cyclin D1 levels in lung homogenates (Figure 4A,C). However, there appears to be a pattern where vasoconstriction increases the reduction in both AMPK and cyclin D1 levels that was induced by colistin in non-septic animals. Namely, AMPK to β-actin levels ratio was 0.94 ± 0.31 in control, 0.57 ± 0.2 in colistin and 0.41 ± 0.005 in colistin + VA, respectively. As far as cyclin D1 to β-actin levels ratios were concerned, they were 1.038 ± 0.34, 0.88 ± 0.23 and 0.63 ± 0.006 in the control, colistin alone or colistin and vasoactive drugs, respectively. This pattern does not seem to be present in LPS-induced sepsis animals, where VA appears to ameliorate the colistin effect (Figure 4A,C). 

On the other hand, caspase 3 levels in the lung homogenates of septic animals treated with colistin were much lower than the respective levels in non-septic animals (** *p* < 0.01, Mann–Whitney test, Figure 4B). These levels were statistically significantly lower in colistin alone-treated animals compared to control or colistin + VA animals (### *p* < 0.001 and # *p* < 0.05 Mann–Whitney test, Figure 4B). Namely, the caspase 3 to β-actin ratio was 0.82 ± 0.06, 0.16 ± 0.01 and 0.61 ± 0.2 in the control, colistin alone and colistin in the presence of VA drugs, respectively (Figure 4B). 

From the above observed results, both sepsis and colistin appear to alter cell death induction, as estimated with caspase 3 evaluation in lung homogenates from emphysematous lungs in the present sepsis model. However, there are no statistically significant alterations in cell metabolism or proliferation, as evaluated using the AMPK and cyclin D1 levels in all animal groups. 

## 3. Discussion

Emphysema is prevalent in various respiratory diseases like COPD and cystic fibrosis. Sepsis stands as a severe and potentially life-threatening condition frequently encountered among these patients when admitted to hospitals’ ICUs globally. Vasoconstrictive drugs are employed in sepsis to help stabilize blood pressure and improve perfusion to vital organs while colistin, a last-resort antibiotic, is a crucial treatment option in such critical scenarios. Our study aimed to evaluate the effects of colistin, combined or not with vasoconstrictive drugs, in emphysematous animals in the presence or absence of septic conditions, focusing on the resulting lung pathophysiology and inflammatory responses. 

Our findings revealed that all animal groups exhibited evident emphysematous changes, affirming the reliability of our emphysema model (Figure 1). Additionally, septic conditions accentuated the extent of lung damage, underscoring the impact of sepsis on exacerbating lung pathology [20,21]. Colistin administration, either alone or in combination with vasoconstrictive drugs, notably stimulated inflammatory cell infiltration, with lymphocytes observed in colistin-treated animals. This demonstrates the potential immunomodulatory effects of colistin, eliciting an increased immune response within lung tissues. This finding aligns with the observation that colistin influences the activity of neutrophils that are recruited in inflamed lung tissue by increasing elastase activity [12].

Surprisingly, vasoconstriction did not significantly influence the effects of colistin or sepsis. Pulmonary vasoconstriction acts as a homeostatic response to sensing hypoxia, ensuring an adequate air supply [22] and as mentioned above, vasoconstrictive drugs are utilized in the ICU to counteract the profound vasodilation commonly observed in sepsis. Our results indicate that vasoconstrictive drugs, while critical in managing sepsis clinically, did not noticeably alter the observed outcomes of lung pathology in our animal models.

Sepsis led to an increase in colistin levels in the lungs, which is indicative of the fact that sepsis alters the distribution of the drug in the tissues. Especially in the lungs, sepsis can induce acute lung injury in the presence of inflammation with the contribution of epithelial cells, macrophages and lymphoid cells [22]. Despite employing a colistin dosage similar to clinical practice to mitigate nephrotoxic effects [23], the narrow therapeutic index of colistin raises the possibility that the elevated colistin levels observed in the lungs of septic animals could also stem, at least partially, from impaired kidney function and reduced drug elimination induced by colistin itself. An intriguing observation was the increase in colistin levels in the lung tissue following vasoconstriction in septic animals. This finding prompts considerations about the interplay between vasoconstrictive drugs and colistin distribution or metabolism within the lungs during sepsis, potentially leading to increased local concentrations of colistin in the lung microenvironment. 

The presence of inflammation in the lung specimens of this study was confirmed by the presence of inflammatory cytokines in these tissues (Figure 3). The differences observed between IL-1β and TNF-α levels in lung homogenates can be representative of the different pathways and/or the different origins of these cytokines. Even though both TNF-α and IL-1β are pro-inflammatory cytokines that induce similar effects and are activated by similar factors regulating mainly their transcription, there are differences in the sensitivity of their response to LPS [24]. Namely, LPS-induced inflammation and/ or sepsis results in the activation of both TNF-α and IL-1β, but these cytokines initiate different pathways’ cascades that cooperate in the induced effect, even though they are distinctively different [24]. 

Colistin, in the presence or absence of vasoconstrictive drugs, does not have any statistically significant effect as far as the levels of AMPK and Cyclin-D1 are concerned (Figure 4). This suggests that neither colistin nor sepsis could affect the metabolism and cell proliferation, respectively, in a way that could be observed by dot blot protein analysis in lung homogenates. AMPK, which is an intracellular serine/threonine kinase, has been found to be a key regulatory factor of cell metabolism that plays an important role, especially in stress response pathways activation, such as autophagy. However, the activation of AMPK has been reported to be important for the handling of mainly viral infections in the lung rather than emphysematous inflammation [25]. Furthermore, lung inflammation is a factor that can lead to cell senescence that results in cell cycle arrest [26] and, therefore, the Cyclin- D1 levels in inflamed lung specimens would not be easy to assess. 

However, the presence of colistin inhibited the activation of caspase- 3 in septic animals, with this effect being ameliorated in the presence of vasoactive drugs (Figure 4). Both emphysema and sepsis are factors that can lead to inflammatory cell infiltration in the lung, which is characteristic of tissue inflammation. This leads to the activation of cascades that result in an imbalance of proteolytic and anti-proteolytic activity in the lung parenchyma, leading to the destruction of lung tissue [27]. However, the activation of the caspases cascade is a complex mechanism that is regulated by many different molecules and is affected by the overall condition of the tissue. 

The major limitation of the present study was the small number of animals that were used. This limitation could be the reason why, even though there were some effects that appeared to be present, they were not statistically significant. However, this study shows that sepsis can affect the inflammation induced by emphysematous damages in the lung and that colistin in the presence of vasoconstriction can affect the inflammatory process and the induction of the activation of signaling pathways responsible for tissue response to such stimuli. 

## 4. Materials and Methods

### 4.1. Animals 

In this study we used 14 adult, 6–8 weeks old, male Wistar rats. All animals were maintained in regulation boxes and given free access to food and water. Furthermore, a 12 h day–night cycle was maintained through the experimental period. The room in the animal house where they were kept had a temperature between 20 and 25 °C, it was also quiet and had 24 h ventilation. All animals used in the study were acclimatized by the researchers since they were responsible for daily care, treatment with normal saline, colistin and colistin combined with VA drugs and conducting the euthanasia and lung harvest. The maximum number of animals in each cage was 3 rats. Every day, prior to treatment, each animal was transferred to a sealed cylindric chamber (~6.280 cm^3^; 20 cm height × 20 cm diameter) with 3 mL of diethyl ether (Merk, Darmstadt, Germany) and remained there for 2–4 min in order to induce emphysema. The animals’ development was monitored by body weight measurement and estimation of the amount of food and water the animals consumed. Their behavior and interactions with the other animals kept in the same cage were observed and described as normal. The study was carried out in strict accordance with the recommendations in the Guide for the Care and Use of Laboratory Animals [28] and institutional guidelines.

### 4.2. Animal Treatment 

There were 5 non-septic and 9 LPS-induced septic animals. The sepsis induction was performed as previously described [15]. More specifically, the sepsis model used included intraperitoneally injection of LPS (Lipopolysaccharides from *Escherichia coli*, 10,000 EU/mg, purified by phenol extraction, Sigma Aldrich, Darmstadt, Germany). In this study, 250 mg/ kg of LPS was administered to the rats in order to make them septic. Sepsis was induced, and after 5 days, the rest of the treatments were administered to the animals. Sepsis induction was confirmed by observing a reduction in weight gain [15]. After this procedure, animals were divided in 3 subgroups randomly. The groups were control animals that received intraperitoneally administered normal saline, colistin rats that were injected with colistin alone (150,000 U/kg/day; Colistin methate Sodium, 1,000,000 IU/vial, Norma Hellas, Athens, Greece) and colistin and vasoconstrictive drugs group that consisted of animals that had been injected with a mixture of colistin (150,000 U/kg/day) and arginine vasopressin (AVP: 4 U/kg/day; Empressin^®^; 1 mg is equivalent to 530 units according to the US Food and Drug Administration) and/or nor-adrenaline (NA: 25 μg/kg/day). All treatments were administered for 10 days prior to euthanasia and tissue harvest. 

### 4.3. Tissues Processing

Euthanasia of animals was conducted using decapitation after sedation with ether. The right lung lobe was harvested after euthanasia. All excessive and neighboring tissues were removed. In most of the specimens, the lungs were filled with blood, an observation indicative of lung injury due to ether inhalation, throughout the experimental procedure. Half of the specimen was used for histopathological evaluation and the other half was snap-frozen after incubation with isopentane for 1 min and then homogenized (Heidolph Silent Cruser S, Heidolph Instruments GmbH & Co. KG, Schwabach, Germany) with 1× PBS. The part that was going to be histopathologically evaluated was fixed with 4% formalin embedding and stored in a vial filed with formalin for no longer than 48 h. The snap-frozen specimen was stored at −80 °C prior to and after homogenization. 

### 4.4. Histopathological Evaluation 

The lungs that were fixed in 4% formalin were dehydrated, embedded in paraffin wax and cut in 5 μm slices. Hematoxylin–eosin (H&E staining) [29] was used for histopathological evaluation. In order to perform the staining, the lung sections were deparaffinized, and then incubated twice in xylene and three times in 100% and 95% ethanol. Afterward, a water wash and hematoxylin staining followed. The differentiation of the tissue slices was performed with the use of an acidic buffer, followed by one wash with water, and incubation in 95% ethanol. Eosin staining, for a maximum period of 1 min, was performed, and three and two washes with ethanol and xylene, respectively, took place. Tissue specimens were mounted in Eukitt^®^ mounting medium (Sigma, Darmstadt, Germany) and observed under a microscope using 20× magnification. 

All slices were evaluated for emphysematous alterations, hemorrhage and immune cell infiltration. 

### 4.5. Colistin Levels Evaluation

The levels of colistin in lung samples from all treatment groups were evaluated using a commercially available ELISA kit, the Col (Colistin) ELISA Kit (Elabscience Biotechnology Inc., Beijing, China), according to the manual’s instructions. More specifically, the same amount of tissue (0.1 g) was homogenized in 1× PBS and used for the ELISA kit. The OD was measured in a photometer (630 nm) and colistin levels were evaluated. A standard curve of known colistin concentrations was used in order to estimate the amount of colistin in each sample, expressed in ppm. 

### 4.6. Cytokine Concentration Evaluation

The levels of IL-1β and TNF-α were evaluated in lung homogenates using ELISA kits as well. More specifically, the IL-1β and the TNF-α ELISA kits (MABTECH, Nacka Strand, Sweden) were used to estimate cytokine production in the samples from the treated animals, according to the manufacturer’s manual instructions. The same amount of lung homogenate was used for each evaluation. Furthermore, a standard curve of fixed cytokine levels was used for concentration evaluation in pg/mL. 

### 4.7. Protein Analysis

Total protein concentration in homogenized tissues was estimated with the use of the Bradford method. More specifically, homogenized tissues were centrifuged in 1000 g, 4 °C for 20 min and then 5 μL of the supernatant was used for total protein measurement. The supernatant was loaded with 200 μL BIORAD solution (Bio-Rad Laboratories Inc., Hercules, CA, USA) and 795 μL H_2_O. A photometer (595 nm) was used for OD measurement. A standard curve made with known concentrations of BSA was used for total protein concentration evaluation. Dot blot analysis was performed using 4 μg of total protein [30]. The antibodies that were used for metabolism, cell death and cell proliferation evaluation were anti-AMPK rabbit monoclonal antibody (1:1000, Cell Signaling Technology Inc., Danvers, MA, USA), anti-caspase-3 rabbit monoclonal antibody (1:1000, Cell Signaling), anti-cyclin-D1 rabbit monoclonal antibody (1:200, Abcam, Boston, MA, USA) and anti-β-actin mouse monoclonal antibody (1:3000, Cell Signaling) followed by ECL. Then, 4 μg of the total protein was loaded on a nitrocellulose membrane using a grid. The transfer of the protein to the membrane was performed due to gravity. Incubation of the membrane with BSA blocks any non-specific binding. Incubation with the secondary HRP-linked antibody followed incubation with the primary antibody. A standard curve that used known concentrations of BSA was used for normalization. The intensity levels of each expressed protein were estimated. All results were presented as detected protein to β-actin levels ratio. β-actin was used since its expression is considered standard and the same regardless of the sample origin or treatment used. Protein level quantification was performed using the “Gel” analysis commands of Image J software 1.54D. 

The antibody selection that was used for signaling pathways’ activation evaluation was performed based on the fact that they represent 3 basic and different cell functions, namely metabolism, cell death and cell proliferation. AMPK is a key protein in energy homeostatic mechanisms that is expressed when ATP levels are low, while deactivation of AMPK leads to dysregulation of cell autophagy [31]. On the other hand, caspase-3 is an enzyme that is activated when cells undergo apoptotic cell death [32,33]. Finally, cyclin-D1 is a protein that regulates the G1 phase of the cell cycle and is activated in proliferating cells [34].

### 4.8. Statistical Analysis

All data presented in the figures were expressed as means ±SEM, and N refers to the number of independent samples. Statistical significance was confirmed using the Mann–Whitney test. A difference was considered significant when *p*  <  0.05. GraphPad Prism software 4.00 was used for statistical analysis, while all image analysis was performed with the Image J software. 

## 5. Conclusions

The findings of this study have potential implications for the treatment of COPD and cystic fibrosis patients diagnosed with sepsis in the ICU. The immunomodulatory effects of colistin observed in this study suggest that colistin might play a role in modulating immune responses within the lungs of these patient populations. The increased colistin levels following vasoconstriction in septic conditions pose questions about the optimization of colistin delivery and distribution in critically ill patients with lung pathologies. Moreover, the impact of colistin in combination with vasoconstrictive drugs on inflammatory markers and apoptosis pathways introduces complexities that need further exploration in the context of COPD and cystic fibrosis patients with sepsis in the ICU. Understanding the intricate interactions of these medications in such critical conditions may guide the development of tailored therapeutic strategies to better manage inflammatory responses and lung pathologies in these patient populations. In conclusion, our study provides valuable insights into the complex interactions between medications, sepsis, and lung pathologies, presenting potential considerations for the use of colistin and vasoconstrictive drugs in COPD and cystic fibrosis patients diagnosed with sepsis in the ICU. In light of these observations, further studies are warranted to evaluate the potential need for colistin dosage reduction in patients with lung pathologies, such as COPD and cystic fibrosis, who are diagnosed with sepsis.

## Data Availability

All data are available upon request sent to the corresponding author.
